# Targeting Discoidin Domain Receptor 1 (DDR1) Signaling and Its Crosstalk with β_1_-Integrin Emerges as a Key Factor for Breast Cancer Chemosensitization upon Collagen Type 1 Binding

**DOI:** 10.3390/ijms21144956

**Published:** 2020-07-13

**Authors:** Fabian Baltes, Julia Caspers, Svenja Henze, Martin Schlesinger, Gerd Bendas

**Affiliations:** Pharmaceutical Institute, University of Bonn, An der Immenburg 4, 53123 Bonn, Germany; fabian-baltes@uni-bonn.de (F.B.); JuleCaspers@web.de (J.C.); svenja.henze@uni-bonn.de (S.H.); martin.schlesinger@uni-bonn.de (M.S.)

**Keywords:** ABC transporter, breast cancer, chemoresistance, collagen, DDR1, EGFR, integrin, MAPK

## Abstract

Collagen type 1 (COL1) is a ubiquitously existing extracellular matrix protein whose high density in breast tissue favors metastasis and chemoresistance. COL1-binding of MDA-MB-231 and MCF-7 breast cancer cells is mainly dependent on β_1_-integrins (ITGB1). Here, we elucidate the signaling of chemoresistance in both cell lines and their ITGB1-knockdown mutants and elucidated MAPK pathway to be strongly upregulated upon COL1 binding. Notably, Discoidin Domain Receptor 1 (DDR1) was identified as another important COL1-sensor, which is permanently active but takes over the role of COL1-receptor maintaining MAPK activation in ITGB1-knockdown cells. Consequently, inhibition of DDR1 and ERK1/2 act synergistically, and sensitize the cells for cytostatic treatments using mitoxantrone, or doxorubicin, which was associated with an impaired ABCG2 drug efflux transporter activity. These data favor DDR1 as a promising target for cancer cell sensitization, most likely in combination with MAPK pathway inhibitors to circumvent COL1 induced transporter resistance axis. Since ITGB1-knockdown also induces upregulation of pEGFR in MDA-MB-231 cells, inhibitory approaches including EGFR inhibitors, such as gefitinib appear promising for pharmacological interference. These findings provide evidence for the highly dynamic adaptation of breast cancer cells in maintaining matrix binding to circumvent cytotoxicity and highlight DDR1 signaling as a target for sensitization approaches.

## 1. Introduction

Breast cancer (BC) is the most common type of malignancy in the female population and represents the second leading cause of death in women among the cancer mortalities. According to statistical evaluations and actual estimates, about 30% of women will develop BC in their lifetime and about 15% of women run into danger to die from that malignancy [1]. BC is a heterogeneous disease with respect to the expression profile of molecular targets such as estrogen receptor, progesterone receptor, or human epidermal growth factor receptor 2 (HER2). The therapeutic treatment of BC with antineoplastic drugs is related to this subtype-selectivity [2]. Triple-negative breast cancer (TNBC) cells do not express any of these three target structures and therefore cannot be treated by targeting them. While hormone-receptor-positive BC is mainly treated by anti-hormonal therapy, classical cytostatic drugs are still used against TNBC despite their side-effects. However, TNBC frequently express epidermal growth factor receptor (EGFR) at a varying extent, which has also been considered as a potential biomarker for TNBC [3,4,5]. Although a significant correlation between EGFR expression and poorer outcomes in TNBC has been shown [6,7], a clinical benefit and advantage of EGFR inhibitors over other treatments could not be derived [8]. BC mortality is mainly caused by a chemoresistant recurrence of the disease after an initial successful treatment or by metastasis of BC. However, both processes are often functionally interlinked.

Resulting from further insight into the role of tumor microenvironment on tumor progression and invasiveness, the impact of the extracellular matrix (ECM) components on chemoresistance and metastatic spread of tumors attracts growing attention. Collagens are a major component of the ECM and there is accumulating evidence confirming the critical role of collagen and especially collagen type 1 (COL1) in the development and outcome of BC [9,10,11]. In particular, increased deposition and reorientation of stromal collagen fibers have been associated with BC progression and invasiveness, often in terms of fibrosis. A functional link between metastasis and ECM protein production by BC cells (BCCs) has been elucidated to that effect that, e.g., collagen enhanced recruitment of platelets to circulating BCCs, and thus promoted lung retention and colonization of cancer cells [12]. Concerning chemoresistance, ECM is reportedly implicated in lowering the response to therapy of BC [13,14,15,16]. Therefore, ECM gene expression patterns can be utilized as a prognostic and predictive value to evaluate the sensitivity towards therapeutics in TNBC [17]. At a functional level, the link between ECM binding and loss of chemosensitivity of BC refers to the phenomenon of cell-adhesion-mediated drug resistance (CAM-DR). CAM-DR has initially been described as a rapid, nongenetic adaptation of leukemia cells to cytostatics for an increased survival of treatment, associated with the phenomenon of minimal residual disease [18,19]. However, in the meantime, CAM-DR has been accepted as a phenomenon of de novo resistance with relevance for different solid tumor cells [20], displaying a prerequisite for the acquisition of genetic drug resistance mechanisms.

Integrins are heterodimeric adhesion receptors that consist of one α-subunit recognizing the binding partner and a β-subunit connected to intracellular kinases that mediate signals into the cells (outside-in). Integrin subunit β_1_ (ITGB1) has been described to possess a key role for mediating adhesion to COL1 since all four COL1-binding integrins consist of ITGB1. Therefore, ITGB1 is described to induce COL1-based CAM-DR in BC and melanoma cells, respectively [21,22]. We have recently reported that COL1 binding of human MCF-7 or MDA-MB-231 BCCs induces the upregulation of ABC efflux transporters, dominantly contributing to the development of a chemoresistance [23]. However, the underlying molecular mechanisms of the COL1/ABC transporter axis remain to be elucidated especially in light of the findings that ITGB1-knockdown (kd) only partially reverses resistance. This opens the view to other potential COL1 binding proteins contributing to CAM-DR.

Discoidin Domain Receptor 1 (DDR1) appears as a highly likely candidate to confer a COL1 binding of cells into an increased tumorigenicity. DDR1 belongs to the family of nonintegrin collagen receptor tyrosine kinases whose deregulation is described in various diseases such as arthritis, fibrosis and the progression of various types of cancer [24]. Aberrant expression of DDR1 has been detected in several human cancers including ovarian, breast, gastric and lung cancers, and is often associated with increased invasiveness [25,26]. DDR1 monomers dimerize and bind to GVMGFO motif of COL1 via their extracellular discoidin domain [27]. Afterwards, DDR1 dimers undergo slow but prolonged autophosphorylation at several tyrosine residues in their intracellular kinase domain, which results in DDR1 clustering similar to the mechanism visible in integrins [28]. Although the reports differ in function on how DDR1 affects breast cancer migratory activities [29,30], DDR1 effects appear to be controlled by interacting partners in the cellular microenvironment. The role of DDR1 in adhesion is controversially discussed. On the one hand, DDR1 has been reported to possess adhesive properties to COL1, in contrast to DDR2 [31]. On the other hand, DDR1 is known to stabilize E-cadherin [32] or COL1 binding function of integrins α_1_β_1_/α_2_β_1_ [33]. Concerning the relationship of reduced sensitivity of BCCs to cytotoxic treatment and DDR1, a pathway via activating p53 was demonstrated to be responsible for increased survival mediated by DDR1 [34]. Others reported on a DDR1-mediated enhanced chemoresistance in BCCs by upregulation of COX-2 via NFkB pathway activation [35]. Although on basis of these and similar findings in other tumor entities [36], DDR1 emerges as a promising novel target to interfere with tumorigenicity, an explicit involvement of DDR1 in the process of CAM-DR is experimentally still lacking. In light of shared signaling pathway activities between integrins and DDR1 [37], the elucidation of a common role of DDR1 and ITGB1, or a dynamic shift in activities between both COL1 binding receptors and the downstream signaling remains a challenging approach. Notably, DDR1 shares several signaling pathways with integrins such as protein kinase B (AKT) or mitogen-activated protein kinase (MAPK) [38].

Here, we provide evidence that COL1 binding in triple-negative MDA-MB-231 BCCs activates MAPK pathway leading to cellular resistance against cytostatics, such as mitoxantrone, partly by ERK1/2-dependent ABC transporter activation. Upon a knockdown of ITBG1, DDR1 takes over the role of COL1 sensor maintaining the COL1 activation of MAPK, emphasizing a crosstalk between integrin β_4_ and receptor tyrosine kinases such as EGFR. Notably, noninvasive and estrogen-receptor-positive MCF-7 BCC, which display an initially higher level of DDR1 partly differ in the underlying signaling mechanisms. This highly dynamic adaptation of malignant tumor signaling argues for a multitarget-inhibition approach, which is shown here to successfully interrupt CAM-DR.

## 2. Results

### 2.1. MAPK Signaling Is Activated by COL1 Binding of Cells, Even in the Absence of ITGB1

Binding of MDA-MB-231 or MCF-7 BCCs to COL1 activates several signaling pathways and even induces resistance against different antineoplastic drugs. Focusing primarily on ITGB1 in these terms, we established ITGB1 knockdown variants, which displayed a downregulation of ITGB1 to an amount of 21% and 7.5%, respectively [23]. However, knockdown of ITGB1 did neither reverse the COL1 induced resistance, nor completely circumvented adhesion to COL1 in both cell lines.

**Figure 1:**
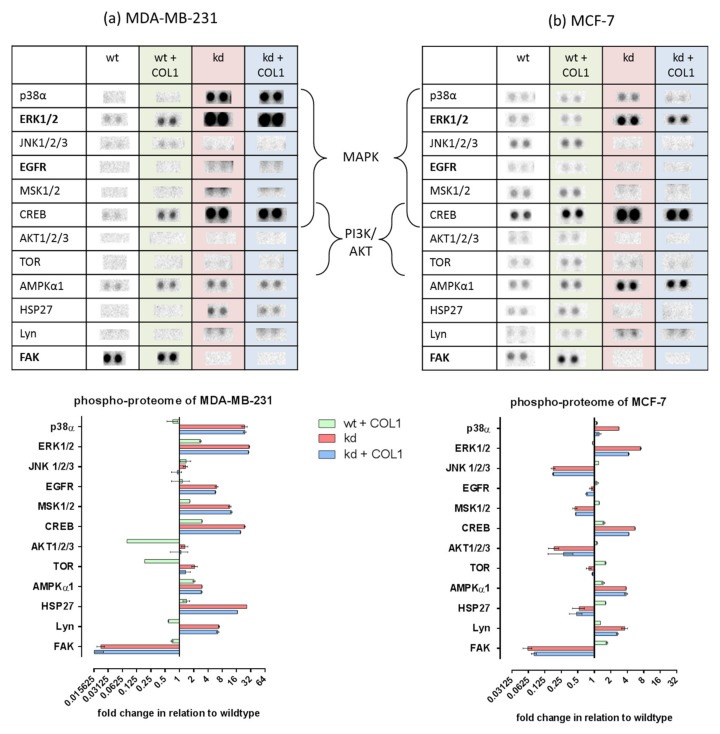
Data of a human phospho-kinase proteome profiler array of MDA-MB-231 (**a**) and MCF-7 (**b**) human breast cancer cells. The impact of collagen type 1 (COL1, green), as well as the absence (knockdown, kd) of ITGB1 (red) and COL1 binding in the absence of β_1_-integrins (blue) in reference to wildtype (wt) cells (black, basis line = 1). The intensities of the phosphorylated kinases were derived by pixel density analysis of the respective arrays shown above and are outlined in a log2 manner (*n* = 1). Highlighted are both main survival pathways mitogen-activated protein kinase (MAPK) and phosphoinositide 3-kinase/protein kinase B (PI3K/AKT).

To provide a first insight into the underlying signaling pathways of the COL1-resistance axis in dependence of ITGB1, we performed a human phospho-kinase proteome profiler array. Untreated and COL1-treated (black and green) MDA-MB-231 (Figure 1a) and MCF-7 (Figure 1b) cells, as well as the kinome of their ITGB1-kd clones in the presence and absence of COL1 (red and blue), were analyzed. In general, the ITGB1-kd led to a decrease of phosphorylated focal adhesion kinase (pFAK, Y397) to a level of 10% in both cell lines. FAK is a classical mediator of integrin signaling since it is directly linked to and activated by ITGB1-subunit. Furthermore, the ITGB1-kd had a stronger consequence for deactivating a greater spectrum of kinases in MCF-7 cells, compared to MDA-MB-231 cells. Interestingly, the Src family kinase Lyn, which is associated with a more aggressive BC phenotype [39], is increased in both cell lines upon ITGB1-kd.

Although PI3K/AKT signaling is the main reason for breast cancer development [40,41], we could not detect any spots or differences in MDA-MB-231 cells upon COL1 or/and ITGB1-kd. In MCF-7 cells, slight basal levels of AKT and mTOR were seen, probably due to a PI3KCA mutation, but these levels were reduced upon ITGB1-kd.

The impact of COL1 in both cell lines is mainly based on an increase in MAPK-dependent kinases, which is more expressed in MDA-MB-231 cells possibly due to their RAS/BRAF mutation [42]. This MAPK activation was indicated by the higher levels of activated p-p38, pERK1/2, pCREB, pP70S6 kinase in both ITGB1-kd cell lines, or pHSP27 only in the case of MDA-MB-231 cells.

However, a difference between the two cell lines refers to the strong activation of EGFR in MDA-MB-231kd cells, which did not appear in the MCF-7kd cells. On that basis, the question emerged in which cellular receptors take over the role of ITGB1 in contact with COL1 shifting the cellular signals into the MAPK pathway.

### 2.2. DDR1 Is Involved in MCF-7 and MDA-MB-231 Cell Adhesion to COL1

**Figure 2:**
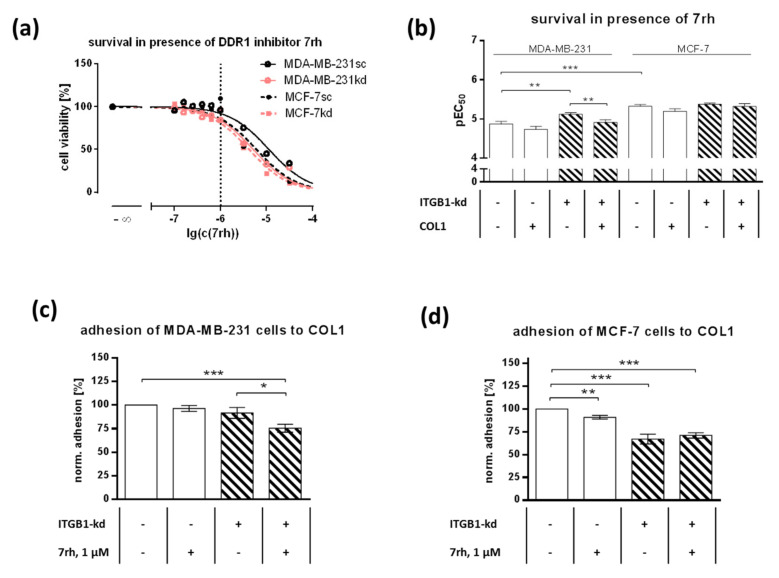
(**a**) Representative survival curves of MDA-MB-231 and MCF-7 cells (scrambled, sc) and their integrin β_1_-knockdown (ITGB1-kd) mutants on collagen type 1 (COL1) in the presence of DDR1-inhibitor 7rh for 72 h. The nontoxic concentration of 1 µM, used for adhesion studies in (**c**,**d**) is marked. (**b**) Statistical analysis of survival pEC_50_ of DDR1-inhibitor 7rh in MDA-MB-231 and MCF-7 scrambled and ITGB1-kd cells in the presence and absence of COL1. Data represent means ± SEM of at least *n* = 11 biological replicates. (**c**,**d**) Adhesion of MDA-MB-231 cells (**c**) and MCF-7 cells (**d**) and their ITGB1-kd mutants on COL1 in the presence or absence of DDR1-inhibitor 7rh. Data represent means ± SEM of *n* = 6 different biological replicates. Statistical analysis was performed via unpaired *t*-test (* *p* < 0.05; ** *p* < 0.01; *** *p* < 0.001).

Based on the literature, DDR1 is the most probable COL1 adhesion receptor besides ITGB1 and also involved in MAPK signaling. DDR1 is known to be expressed in MCF-7 cells to a high and in MDA-MB-231 cells to a low degree [43]. To focus the role of DDR1, we applied the selective small-molecule DDR1-inhibitor 7rh, which should possess anti-adhesive effects by blocking the intracellular ATP binding site of DDR1 and therefore possibly suppress adhesion crosstalk [44,45]. At first, we investigated the cytotoxicity of 7rh in both cell lines and the indicated ITGB1-kd subtypes (Figure 2a,b). Notably, MCF-7sc cells possessed a significant higher sensitivity (*p* < 0.0001) comparing the EC_50_ values (pEC_50_ = 5.325 ± 0.046; 4.73 µM) to MDA-MB-231sc cells (pEC_50_ = 4.875 ± 0.067; 13.34 µM), obviously related to the higher DDR1 level in MCF-7 cells mentioned above. Furthermore, both ITGB1-kd variants displayed a higher sensitivity towards DDR1-inhibition compared to their corresponding control cells, which can be explained by the higher impact of DDR1 on cell behavior upon ITGB1-kd. In the case of MDA-MB-231 cells, the difference between sc (pEC_50_ = 4.875 ± 0.067; 13.34 µM) and kd (pEC_50_ = 5.123 ± 0.039; 7.53 µM) was significant (*p* = 0.0033). It also became evident that in the presence of COL1, independently of ITGB1 status, cells could tolerate higher concentrations of 7rh cytotoxicity, especially visible in MDA-MB-231kd cells (*p* = 0.0075).

Using 1 µM as a nontoxic concentration of 7rh, the impact of DDR1 on cell adhesion to COL1 was detected in the dependence of ITGB1 status. ITGB1-kd had only a minor impact on reducing MDA-MB-231cell adhesion. 7rh hardly affected adhesion of MDA-MB-231sc cells (92%), but induced reduction from 92% to 76% in the ITGB1-kd variant (*p* = 0.0474, Figure 2c). In contrast, the knockdown of ITGB1 impaired the adhesion to COL1 by 33% (*p* < 0.0001) in MCF-7kd cells (Figure 2d). The anti-adhesive properties of 7rh were significant in MCF-7sc cells reducing the adhesion from 100% to 91% (*p* = 0.0015), while 7rh has no further effects in MCF-7kd cells. These data indicate the role of DDR1 in both cell lines, which becomes more evident in MDA-MB-231 cells upon ITGB1-kd.

### 2.3. ITGB1-DDR1 Crosstalk Includes MAPK Signaling

To further focus on the cellular function of DDR1 in terms of the COL1 resistance, we checked the cellular expression of DDR1 and pDDR1 upon COL1 binding and/or 7rh treatment in the sc and ITGB1-kd cells by Western blots. Since the proteome arrays indicated the involvement of MAPK signaling, we further elucidated whether DDR1 can induce MAPK signaling. Therefore, we analyzed ERK1/2 phosphorylation upon a blockade of DDR1 using 7rh.

**Figure 3:**
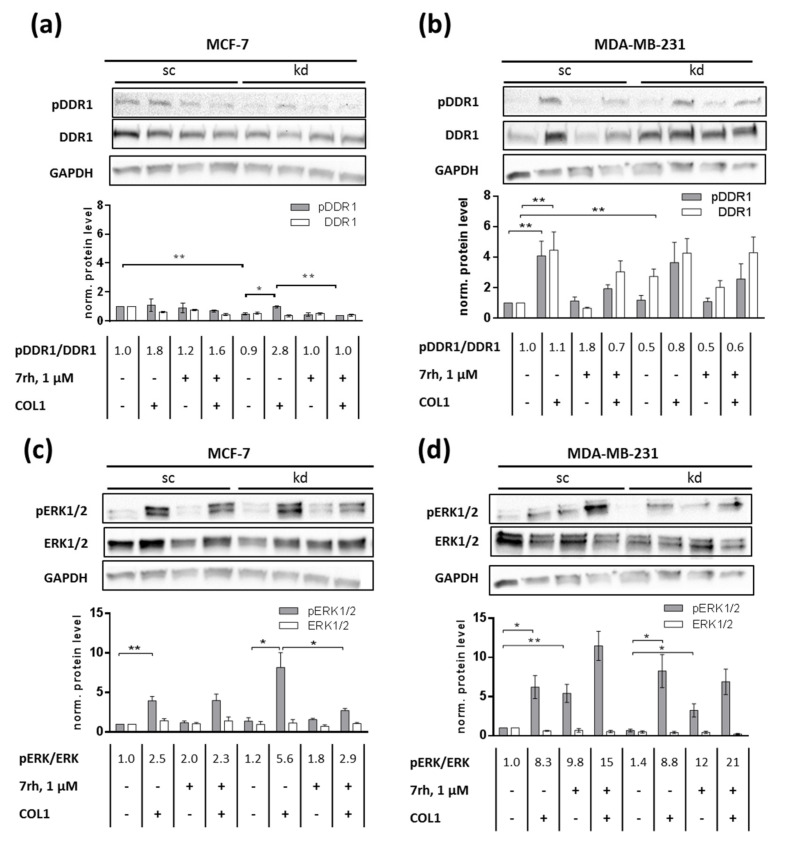
The role of Discoidin Domain Receptor 1 (DDR1) as a collagen type 1 (COL1) receptor in MCF-7 (**a**) and MDA-MB-231 (**b**) (scrambled, sc) cells upon integrin β_1_-knockdown (kd) for the induction of COL1-mediated signaling via ERK1/2 (**c**,**d**). DDR1 is significantly increased in MDA-MB-231kd cells. Western blot data also indicate that DDR1 is activated by COL1 in both cell lines and DDR1 inhibition by 7rh attenuates pDDR1 and pERK1/2 in MCF-7kd cells. In contrast, DDR1-inhibition by 7rh increased pERK1/2 in MDA-MB-231 cells. GAPDH was used as loading control. Data represent means ± SEM of at least *n* = 3 biological samples, statistical analysis was performed using unpaired *t*-test (* *p* < 0.05; ** *p* < 0.01).

MCF-7sc cell binding to COL1 induced DDR1 phosphorylation, which was slightly antagonized by the DDR1-inhibitor 7rh. Notably, in the absence of ITGB1, the MCF-7kd cells reacted more intensive to COL1 binding by a significantly (*p* = 0.0164) stronger DDR1 activation, which was completely (*p* = 0.023) reverted by 7rh (Figure 3a). 

In MDA-MB-231 cells the DDR1-inhibitor reduced the phosphorylation of DDR1 upon COL1 binding as well (Figure 3b). Considering the total amount of DDR1, it was clearly visible that MCF-7 cells reduced this protein upon COL1 stimulus or in the case of ITGB1-kd, whereas MDA-MB-231 cells significantly (*p* = 0.0077) increased the total amount of DDR1 up to 3-fold as a reaction to integrin knockdown. Notably, these findings explain and reflect the adhesion studies shown above (Figure 2c,d) indicating that DDR1-inhibition by 7rh had the strongest impact in those cells with the highest DDR1 levels, namely in MCF-7sc and MDA-MB-231kd cells.

The presence of COL1 significantly increased pERK1/2 in MCF-7sc (*p* = 0.0058, Figure 3c) and MDA-MB-231sc (*p* = 0.0126, Figure 3d) cells. This COL1-mediated increase in MCF-7sc was not antagonized by 7rh. In contrast, in MCF-7kd cells, 7rh was able to reduce pERK1/2 significantly (*p* = 0.0444), which clearly indicates a MAPK activation due to DDR1 binding to COL1. For MDA-MB-231sc and MDA-MB-231kd cells, a correlation between DDR1 activity and ERK1/2 activation was not detectable. On the contrary, coincubation with 7rh significantly increased ERK1/2 phosphorylation eightfold in MDA-MB-231sc (*p* = 0.0089) and kd (*p* = 0.0238) cells. This indicated a potential escape strategy of MDA-MB-231 cells to cope with the DDR1 inhibition.

In summary, these data suggest that DDR1 led to a direct activation of MAPK signaling in MCF-7 cells while MDA-MB-231 cells react to DDR1 inhibition by phosphorylation of ERK1/2.

### 2.4. MAPK Pathway Is Crucial for COL1-Mediated Signaling and Can Be Interfered by ERK1/2-Inhibitor SCH772984

**Figure 4:**
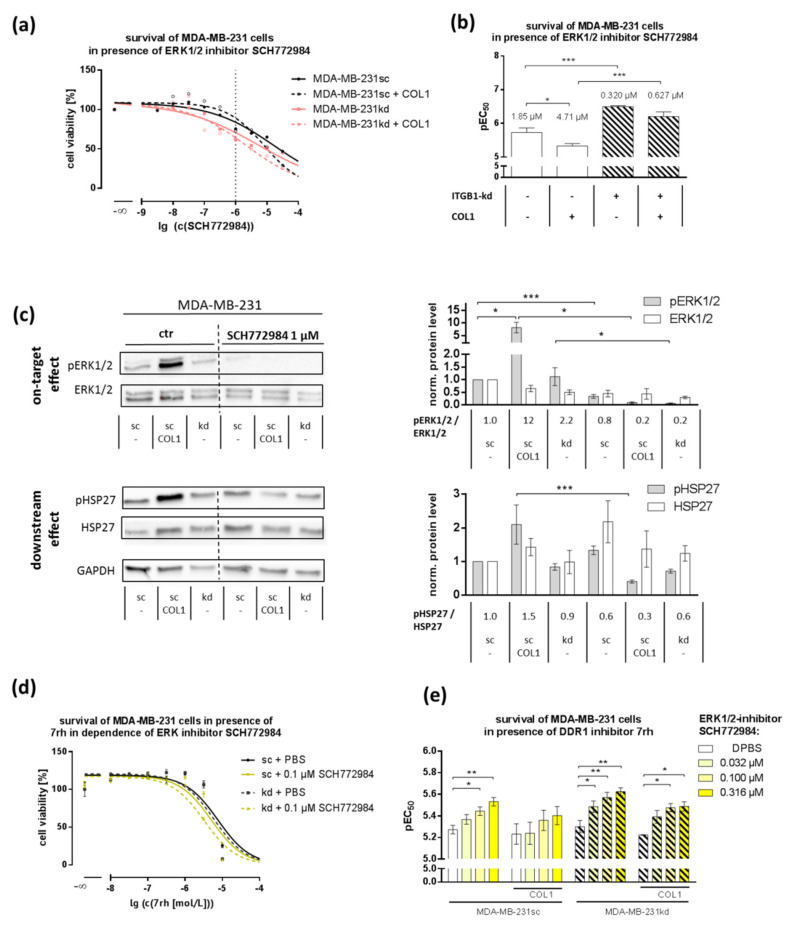
(**a**) Representative survival curves of MDA-MB-231 cells in the presence of ERK1/2-inhibitor SCH772984 for 72 h revealed a higher sensitivity of the integrin β_1_-knockdown (ITGB1-kd) cells towards ERK1/2-inhibition compared to scrambled (sc) cells and a higher resistance upon collagen type 1 (COL1) binding, indicated by the statistical analysis on these cytotoxicity data shown in (**b**) (*n* = at least 5 biological samples). (**c**) Western blot analysis of pERK/ERK and pHSP27/HSP27 with GAPDH as loading control after incubation with ERK1/2–inhibitor SCH772984 in MDA-MB-231 cells (*n* = at least 3 biological samples). (**d**,**e**) Combination effect of 7rh and SCH772984 cytotoxicity in MDA-MB-231 cells shows the pEC_50_ values of Discoidin Domain Receptor 1 (DDR1) inhibitor 7rh cytotoxicity in the dependence of SCH772984 (*n* = at least 3 biological samples). MDA-MB-231 cells display a significant increase in pEC_50_. Statistical analysis was performed via unpaired *t*-test (* *p* < 0.05; ** *p* < 0.01; *** *p* < 0.001).

In order to confirm that ERK1/2 is a key molecule representing the MAPK signaling pathway in integrin and nonintegrin-based COL1-mediated cell binding, we analyzed the cell survival in the presence of the ERK1/2-inhibitor SCH772984. Notably, the ITGB1 knockdown in MDA-MB-231 cells induced a six-fold higher sensitivity towards this inhibitor compared to the scrambled cells in the absence (0.32 µM vs. 1.85 µM, *p* = 0.0004, Figure 4a,b) or presence of COL1 (0.627 µM to 4.710 µM, *p* = 0.0005) indicating a stronger shift to MAPK in the absence of ITGB1. COL1 binding significantly reduced the cytotoxicity in MDA-MB-231sc (1.852 µM to 4.705 µM, *p* = 0.027) and to a lesser extent in ITGB1-kd cells (0.320 µM to 0.627 µM). The impact of ERK1/2-inhibition on MDA-MB-231 viability upon COL1 binding and ITGB1-kd was verified by Annexin V/PI apoptosis assays (Supplementary Figure S1).

ERK1/2-inhibition by SCH772984 at a concentration of 1 µM for 24 h completely suppressed COL1-mediated activation of pERK1/2 and partly its downstream targets, such as pHSP27 [46,47] (Figure 4c), which were elevated in the proteome profiler array (Figure 1a). These data confirm the role of the MAPK pathway in these terms. 

To combine the role of DDR1 as a COL1 sensor and the MAPK pathway represented by ERK1/2 for inhibitor approaches, we investigated the combinatory effects of DDR1- and ERK1/2-inhibitors (Figure 4d,e). A synergistic (CI < 1) effect of DDR1 and ERK1/2-inhibition was evident in MDA-MB-231 cells and even more pronounced in MDA-MB-231kd cells. This synergy is potentially based on the upregulation of pERK1/2 in response to the DDR1-inhibitor 7rh (Figure 3d) thus making interference with pERK1/2 an attractive approach. This synergy was not detectable in MCF-7 cells (Figure S2).

Together, these dates confirm the role of DDR1 as a COL1 cell sensor translating the cell binding into a MAPK activation exhibiting DDR1 and MAPK as potential targets for cytotoxic treatments.

### 2.5. Combination of DDR1- or ERK1/2-Inhibitors with Cytostatics Displays Dose-Dependent Effects

**Figure 5:**
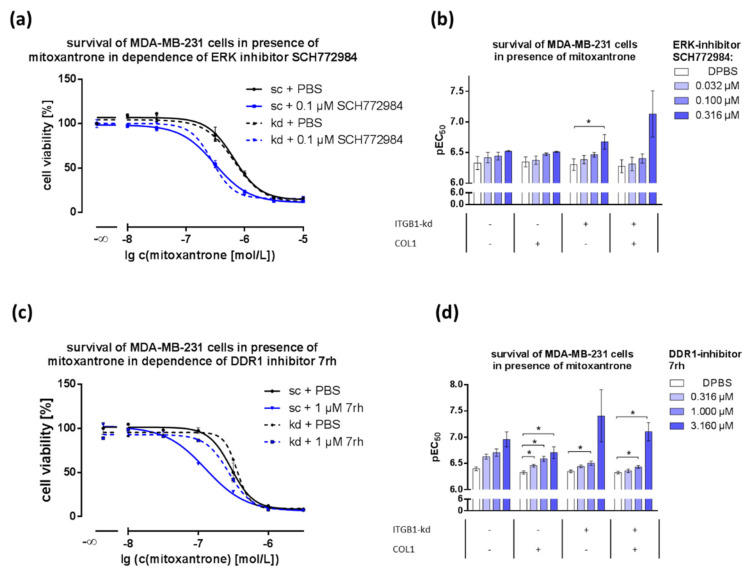
The impact of DDR1- or MAPK-inhibitors on the survival of MDA-MB-231 and MCF-7 cells with mitoxantrone in combinatorial approaches. The ERK1/2-inhibitor SCH772984 (**a**,**b**) and DDR1-inhibitor 7rh (**c**,**d**) were applied at increasing concentrations in dependence of collagen type 1 (COL1) in scrambled (sc) and integrin β_1_-knockdown (ITGB1-kd) cells. The detected increased value of pEC_50_ (*n* = 3) indicates a decrease of EC_50_ values. Statistical analysis was performed via unpaired *t*-test (* *p* < 0.05, *n* = at least 3 biological samples).

In a recent study, we elucidated that COL1 binding of MCF-7 and MDA-MB-231 cells increases the resistance against the breast cancer guideline based cytotoxic agents doxorubicin and mitoxantrone [23]. Here, we demonstrate the role of DDR1 for ERK1/2 activation upon COL1 binding and the promise of both targets for a single or combined approach. Since cytostatics are mostly used in breast cancer entities without hormonal receptors, we focused on MDA-MB-231. Therefore, we sought to investigate the impact of DDR1-inhibition or inhibition of MAPK components (ERK1/2, MEK1/2) on the sensitivity to mitoxantrone. In a combinatorial approach (Figure 5), the impact of COL1 binding and ITGB1-knockdown in MDA-MB-231 cells on sensitivity towards mitoxantrone-mediated cytotoxicity is illustrated. Cells got more sensitive to mitoxantrone with increasing concentration of ERK1/2 inhibitor SCH772984 and 7rh. However, these effects were less evident in MCF-7 cells (Supplementary Figure S3a,b) and in the case of doxorubicin treated MDA-MB-231 cells (Supplementary Figure S3c,d). MDA-MB-231 cells (Figure 5a,b) displayed a strong sensitization towards mitoxantrone in the presence of SCH772984 that was even more pronounced in the ITGB1-kd variant. Whereas COL1 showed no further effect in MDA-MB-231 cells, MCF-7 cells exhibited a sensitization only in the presence of COL1 (Supplementary Figure S3a). In order to validate that effect of SCH77984 in MDA-MB-231 we additionally used U0126 as another ERK1/2-inhibitor (Supplementary Figure S3e,f). In contrast to SCH772984, which allosterically inhibits pERK1/2 itself, U0126 reduces the function of ERK1/2 activating kinase MEK1/2. Although both molecules differ in chemical structure, the resulting effects were quite similar, exposing the combination of mitoxantrone and ERK1/2-inhibition as a promising approach especially in the presence of COL1 and in the absence of ITGB1.

Additionally, the combination of DDR1-inhibitor 7rh and mitoxantrone demonstrated additive effects as well, indicated by significant sensitization effects in MDA-MB-231 cells (Figure 5c,d). The effect was most visible in sc cells and became slighter in kd cells. In contrast, MCF-7 cells displayed this sensitizing effect only in the case of the ITGB1-kd variant (Supplementary Figure S3b).

### 2.6. Synergistic Effect Is Partly Dependent on MAPK Regulated ABC Transport Protein Involvement

**Figure 6:**
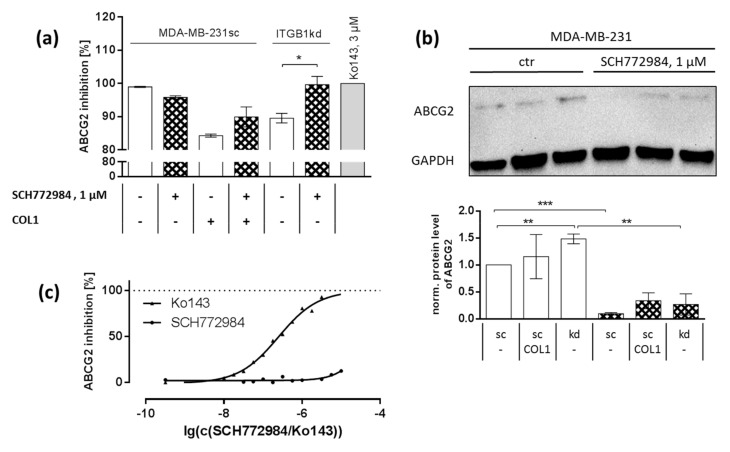
The ABC drug efflux transporter ABCG2 is upregulated by collagen type 1 (COL1) via the MAPK pathway in MDA-MB-231 cells. (**a**) Functional ABCG2 activity assay using the ABCG2 substrate pheophorbide A and its inhibition by SCH772984 which is visible in scrambled (sc) cells in presence of COL1 and in integrin β_1_-knockdown (ITGB1-kd) cells The effect of 24 h incubation of SCH772984 (1 µM) was shown to reduce expression of ABCG2 in sc, COL1-treated and ITGB1-kd cells in Western blot experiments using GAPDH as loading control (**b**) (in both figures *n* = at least 3 biological samples). (**c**) Analysis of the inhibitory properties of SCH77284 itself in ABCG2 overexpressing MDCK II BCRP cells revealed no intrinsic inhibitory effect in comparison to BCRP inhibitor Ko143 at all up to the concentration of 1 µM used (n = at least 3 biological samples). Statistical analysis was performed via unpaired *t*-test (* *p* < 0.05; ** *p* < 0.01; *** *p* < 0.001).

The chemoresistance of MCF-7 and MDA-MB-231 cells upon cultivation on COL1 has recently been shown by our group to be partly dependent on a subsequent upregulation of ABC drug efflux transporters such as ABCG2 or ABCB1 [23]. Both transporters can reduce the intracellular levels of cytostatics. Mitoxantrone is mainly exported by ABCG2 and is to a lesser extent a substrate of ABCB1. To follow the COL1/transporter axis taking the impact of ERK1/2-inhibition on chemosensitivity into account, we aimed to elucidate whether ERK1/2-inhibition affects transporter activity. In a first step, we analyzed the impact of a 24 h cell pretreatment with SCH772984 (1 µM) on the functional activity of ABCG2 (Figure 6a) using fluorescent indicator substrates. As previously shown [23], the presence of COL1 and the downregulation of ITGB1 both increased the efflux activity of ABCG2 in MDA-MB-231 cells. This was confirmed here, considering the data in Figure 6a. Notably, these effects were compensated by the ERK1/2-inhibitor SCH772984, most pronounced for the ITGB1-kd (*p* = 0.0253) cells presenting ERK1/2 as a functional link in the COL1/transporter axis. However, these effects were even more visible in the protein expression data of ABCG2 (Figure 6b), showing a significant upregulation of ABCG2 in knockdown cells and a significant (*p* < 0.0001) downregulation of ABCG2 by ERK1/2-inhibition.

In order to exclude that SCH772984 acts directly as an inhibitor of ABCG2, we performed functional transporter assays using an ABCG2 overexpressing cell lines MDCK II breast cancer resistance protein (BCRP) taking Ko143 as standard for maximal inhibition (Figure 6c). For this approach, SCH772984 was added directly to the cells before performing the assay, thus effects by ERK1/2-inhibition could be excluded. No inhibitory potential of SCH772984 towards ABCG2 activity up to the maximal concentration was detectable. In summary, this shows the MAPK dependency of ABCG2 that can be impaired by ERK1/2-inhibitor SCH772984.

### 2.7. ITGB1-Knockdown Is Compensated by Upregulation of ITGB4 and pEGFR

**Figure 7:**
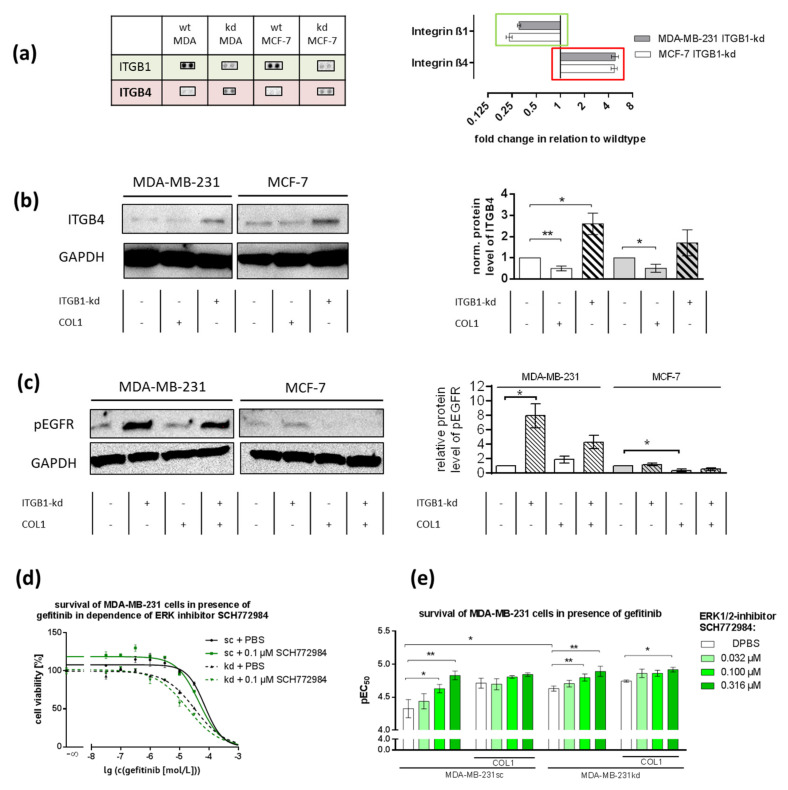
The role of EGFR in MCF-7 and MDA-MB-231 cells in COL1 induced cell activation affected by integrin β_1_-knockdown (ITGB1-kd). (**a**) Data of a surface receptor proteome profiler array indicate that the knockdown of ITGB1 (green) in MDA-MB 231 and MCF-7 cells was antagonized by the upregulation of integrin β_4_ (ITGB4, red) compared to wildtype (wt). (**b**) Western blot of ITGB4 in MCF-7 and MDA-MB-231 scrambled (sc) cells in dependence of collagen type 1 (COL1) or ITGB1-status (*n* = 3 biological samples). GAPDH was used as loading control. Data indicate that ITGB1-kd is associated with the upregulation of ITGB4. ITGB4 can form an active crosstalk to activate (**c**) pEGFR in MDA-MB-231kd, but only slightly in MCF-7kd cells (*n* = 3 biological samples). (**d**,**e**) Blocking of pEGFR by gefitinib was highly synergistic with ERK1/2-inhibition in MDA-MB-231 cells indicating a key role of EGFR in COL1-induced activation of MDA-MB-231 cells (*n* = at least 3 biological samples). Statistical analysis was performed via unpaired *t*-test (* *p* < 0.05; ** *p* < 0.01).

To identify further receptors that could compensate for ITGB1-absence besides DDR1, we performed a proteome profiler array focusing on adhesion receptors. ITGB1-knockdown was proven by missing ITGB1-dots on the membrane, while integrin β_4_ (ITGB4) expression was remarkably (>fourfold) increased (Figure 7a). This effect was verified by Western blot analysis (Figure 7b). Integrin β_4_ (ITGB4) is unable to bind to COL1 but has been described to mediate cell-cell-contacts as a hemidesmosome and to induce an active crosstalk with growth factor receptors, e.g., EGFR [48,49]. Concerning the upregulated pEGFR levels in MDA-MB-231kd cells shown in Figure 1, we therefore explicitly checked for pEGFR in both cell lines by Western blot (Figure 7c).

In the case of MDA-MB-231kd cells, a clear upregulation of pEGFR was detectable. In MCF-7kd cells, the upregulation was only moderate. These data indicate that the functional shift from ITGB1 to DDR1 mediating a COL1 binding additionally triggered an EGFR stimulation in the case of MDA-MB-231 cells contributing to MAPK pathway signaling. Probably, this is induced by an ITGB4 crosstalk. 

These increased pEGFR activities were further elucidated by cytotoxicity approaches, taking gefitinib as a selective inhibitor of EGFR phosphorylation. In order to find a synergistic correlation with other inhibitors used above, we combined the EGFR-inhibitor gefitinib with the ERK1/2-inhibitor SCH772984. In MCF-7 cells the combination of gefitinib with the ERK1/2-inhibitor did not display a synergistic effect confirming a minor role of EGFR in these cells (Supplementary Figure S4a). In contrast, MDA-MB-231sc cells were impressively affected by this combination. Especially in the case of MDA-MB-231kd a sensitization towards gefitinib itself (*p* = 0.0269) and strong synergy with ERK1/2 inhibitor SCH772984 effect was visible (Figure 7d,e, CI < 1).

Furthermore, synergistic combinations of gefitinib with mitoxantrone or doxorubicin were evident in MDA-MB-231 but not in MCF-7 cells (Supplementary Figure S4b–d). In contrast, no effects were seen in the combination of gefitinib with 7rh (data not shown), which indicated the central role of ERK inhibition.

## 3. Discussion

The tumor microenvironment is regarded as a key factor to affect all stages of tumor development and progression and has also been recognized as a fundamental issue to mediate therapy resistance of tumors. Besides cellular host components such as fibroblasts, mesenchymal stem cells, or tumor-associated macrophages that promote tumor growth and metastasis [50,51], embedding and binding of tumors to ECM components appear crucial for attenuating sensitivity to cytotoxic therapy. Referred to as ‘environmental’ or ‘cell adhesion-mediated drug resistance’, these nongenetic de novo resistance processes appear as initial on-set with a general importance for later resistance formation [18]. Nevertheless, CAM-DR has attracted relatively little attention during the last decade despite growing evidence concerning the relevance of CAM-DR for different tumor entities. In vitro simulations of tumor cells grown on isolated ECM components can be regarded as a useful model to get an insight into the cellular consequences of ECM binding and resistance formation thus offering novel targets for sensitization. Here, we follow this approach for breast cancer cells and show, on the one hand, the highly dynamic adaptation of cells to maintain the matrix binding properties using a crosstalk between various adhesion receptors and the resulting survival signaling pathways associated with promising therapeutic targets on the other hand.

Integrins are the most prominent cellular adhesion receptors responsible for contact formation with ECM components. Consequently, ITGB1 activity has been shown to be dominant to confer CAM-DR to tumor cells of different origin upon binding to COL1 [21,22]. Here, we provide evidence that upon ITGB1-kd, COL1 binding capacity is kept active by another important COL1 sensor, namely DDR1. Although both breast cancer cells in this study react differently to the ITGB1-kd with respect to DDR1, either by its upregulation (MDA-MB-231) or a slight downregulation (MCF-7), both cell lines confer COL1 binding into an active DDR1-mediated signaling via the MAPK pathway. This difference in DDR1 expression is also reflected by the cellular reaction towards DDR1-inhibition. Whereas a correlation between DDR1 activity and phosphorylation of ERK1/2 is shown in MCF-7kd cells, MDA-MB-231sc and kd cells react by upregulating pERK1/2. Although this upregulation seems contradictory, Lu et al. observed similar upregulations in the presence of 7rh in nasopharyngeal carcinoma cells (CNE2) [45]. The DDR1-inhibitor increased phosphorylation of Src leading to increased phosphorylation of AKT and MEK1/2. The escape mechanism of MDA-MB-231 cells to upregulate pERK1/2 in reaction to DDR1-blockade offers a new combination strategy for inhibition of MAPK pathway. The reason behind the cell-type-dependent pERK1/2 deregulation remains to be elucidated but could lie in a p53-mediated mechanism since MCF-7 cells contain wild type p53 and MDA-MB-231 express a mutated p53 [34].

DDR1 consists of different splicing variants of which only one is active and contains an additional juxtamembranous kinase domain (DDR1b), which contains Y513 analyzed here by Western blots. This phosphorylation site is a connection of DDR1 to SHC1 (ShcA), which can activate Grb2/SOS and lead to MAPK signaling [27]. Grb2/SOS is also accessible by integrins and EGFR either directly or via SHC1. Therefore, MAPK signaling connects the investigated receptors. The inhibitor 7rh binds to DDR1 in this juxtamembranous area and can probably directly interact with MAPK signaling.

Our data show that DDR1 appears active in COL1 binding and adhesion even in the presence of ITGB1 sharing the MAPK pathway with this integrin. Inhibition of DDR1 sensitized both scrambled control and ITGB1-kd cells for, e.g., mitoxantrone cytotoxicity most likely via an attenuated ERK1/2 activity. Since it is known that ABC transporters depend on ERK1/2 signaling [52], we explicitly checked for this ERK1/2 dependency in MDA-MB-231cells and confirmed that for ABCG2. This offers the DDR1/MAPK axis as a target for sensitization, which is even more promising in a synergistic approach. The inhibition of DDR1 could likely be more advantageous compared to ITGB1-blockade, which has been recognized as a complicated target and failed in recent clinical trials, obviously due to the ubiquitous expression and functionality of this integrin in cells [53].

The role of DDR1 is versatile depending on the tissue. In the case of breast cancer, DDR1 possesses partly contradictory roles related to the specific breast cancer type [54], which is also reflected in this model comparing MCF-7 as an invasive ductal carcinoma with MDA-MB-231 as a model for TNBC. This contradictory role is displayed by the different signaling regulation by DDR1-inhibition. Despite different DDR1 expression in these cells, the function as a negative regulator in terms of EMT is described for both cell lines [55]. DDR1 is also reported to be involved in fibrotic modeling and cancer progression [56]. Notably, DDR1 has been associated with chemoresistance in breast cancer [35], but also ovarian cancer [57] or lymphatics [58].

Concerning DDR1-inhibition, only a few selective DDR1-inhibitors have been developed such as 7rh, with a nanomolar affinity and relative selectivity compared to DDR2-inhibition [44,59]. Notably, several classical RTK-inhibitors were found to unselectively inhibit DDR1 and DDR2, e.g., imatinib, nilotinib, and dasatinib [60]. Thus, it can be assumed that DDR1-inhibition has been a nonrecognized beneficial side-effect in various treatment approaches with RTK-inhibitors in the past. Concerning targeted approaches to block DDR1 aiming to sensitize tumor cells, Aguilera et al. have demonstrated that 7rh reduced DDR1 signaling up to 500 nM in a time-dependent manner in vitro in pancreatic cells and increased response to gemcitabine in vivo in a xenograft model [61]. Our data are in line with these findings showing a sensitization of both cell lines for mitoxantrone and doxorubicin by 7rh-mediated DDR1 blockade.

However, the novelty here is to introduce DDR1 signaling as a potential target to sensitize the cells for both classical cytostatics and targeted kinase inhibitors. It appears remarkable that upon ERK1/2-inhibition, both cell lines increase their sensitivity to the indicated cytostatics, e.g., by reversing the activation of ABCG2, which has recently been indicated as a reason for COL1 induced resistance [23]. Further promising approaches refer to combined inhibition of two or even three signaling constituents. In these terms, the strong upregulation of ITGB4 as another consequence of ITGB1-kd reveals a further important target in MDA-MB-231 cells, namely EGFR signaling. The role and importance of ITGB4 in the development of breast tissue especially in combination with ITGB1 and RTKs are described, so a switch from ITGB1 to ITGB4 is physiologically plausible [62]. Hou et al. showed a similar increase in ITGB4 and pEGFR in a CRISPR-mediated ITGB1-knockout in MDA-MB-231 cells [63]. Our present work extends these findings to an ITGB1 knockdown clone, the availability of COL1 and shows a similar effect in MCF-7 cells. Notably, in MCF-7 cells, the COL1/DDR1 axis directly activates the MAPK pathway. MDA-MB-231 cells induce a crosstalk via EGFR activation under these conditions. A crosstalk between DDR1 and RTKs such as HER2 has been described, but not for EGFR [64]. Consequently, blocking of EGFR activity by gefitinib together with ERK1/2-inhibition or doxorubicin and mitoxantrone resulted in a remarkably increased sensitivity of MDA-MB-231 cells.

Combination therapies are a current topic in chemoresistance research to increase cytotoxicity in cancer cells and to prevent the occurrence of resistance mechanisms. Promising approaches are for example the combinations of Src-inhibitor dasatinib with EGFR-inhibitor afatinib in TNBC [65] or DDR1-inhibitor 7rh in nasopharyngeal carcinoma [45]. Although the combination of ERK1/2 and EGFR blockade in breast cancer has been demonstrated before using MEK1/2-inhibitors [66,67], combination in the context of CAM-DR or COL1 dependency have not been described so far. In this work, we identified two combinations of kinase inhibitors that work selectively in TNBC MDA-MB-231 cells, namely the combination of DDR1-inhibitor 7rh with either ERK1/2-inhibitor SCH772984 or EGFR-inhibitor gefitinib. Since both combinations show better sensitization effects in the presence of COL1, they should be tested in vivo in tumors with high collagenous environments.

Together, COL1 binding of BCCs induces a highly dynamic resistance signaling based on an active crosstalk between ITGB1 and DDR1 as well as ITGB1 and EGFR sharing the MAPK pathway, which offers novel targets to overcome resistance formation. Although ERK1/2, EGFR, or even DDR1 are known as targets to interfere with cancer development and malignancy in oncology for many years, the combined inhibition of these targets offers a novel treatment option in a synergistic manner, especially relevant for TNBC cells.

## 4. Materials and Methods

### 4.1. Cell Culture

The human estrogen-positive MCF-7 BCC line and TNBC line MDA-MB-231 were cultivated in DMEM supplemented with penicillin (10 IU/mL), streptomycin (100 μg/mL), L-glutamine (2 mM) and 10% FBS (all from Pan Biotech, Aidenbach, Germany) in a humidified atmosphere at 37 °C containing 5% CO_2_. In the case of MDA-MB-231 cells, 1 mM sodium pyruvate (Thermo Fisher Scientific Inc, Waltham, MA, USA) was added. Cells were detached using a solution of EDTA (0.2 g/L EDTA × 4 Na) for 10 min at 37 °C. All reagents were from Thermo Fisher Scientific. Mycoplasma contamination was routinely excluded by PCR and cell identity was confirmed by small tandem repeat analysis. Detached cells were counted by CASY cell counter (Omni Life Science, Bremen, Germany) and seeded out to new flasks or plates. The cells were transfected by lentiviral ITGB1-knockdown inducing shRNA (h) particles (Santa Cruz Biotechnology, Heidelberg, Germany, sc-35674-V) with control shRNA (sc-108080) to knockdown ITGB1 as described recently [22,23] with remaining ITGB1 levels of 7.5% (MDA-MB-231kd) or 21% (MCF-7kd) [23]. The transfected cells were further incubated in fresh medium containing 0.2 μg/mL puromycin (Carl Roth GmbH, Karlsruhe, Germany) until 90% confluence and then passaged for further experiments. Knockdown was confirmed by Western blot and flow cytometry using a mouse anti-ITGB1 antibody (Santa Cruz, P2D5).

### 4.2. Coating

Cell culture flasks and microplates were coated with 10 µg/cm^2^ COL1 (Corning, Thermo Fisher Scientific) using a solution of DPBS and the indicated ECM. The flasks/plates were incubated for 4 h at 37 °C, supernatant was aspired and washed with DPBS before filling with medium.

### 4.3. Cell Adhesion

Wells were coated with COL1. After incubation with BSA solution (1%) for 1 h, 20,000 cells were seeded to a total volume of 100 µL medium per well and supplemented with DPBS, 45 min at 37 °C and 5% CO_2_. Afterwards, wells were gently washed to remove nonadherent cells. Adherent cells were fixated by formaldehyde (3%, Merck, Darmstadt, Germany) and stained with methylene blue solution (1%, Merck). After washing with DPBS, cells were lysed using 0.1 M HCl and methylene blue was quantified at 620 nm using a plate reader (Thermomultiscan EX, Thermo Fisher Scientific Inc).

### 4.4. Cell Viability

MTT 3-(4,5-dimethylthiazol-2-yl)-2,5-diphenyltetrazolium bromide (BioChemica, Applichem GmbH, Darmstadt, Germany) was used to measure cell viability. 2000 MCF-7 or 4000 MDA-MB-231 cells were seeded in triplicates at a total volume of 100 µL per well of 96-well plates (Sarstedt AG, Nümbrecht, Germany) either coated with COL1 or left uncoated. Next day, cells were supplemented with dilution series of either DDR1-inhibitor 7rh (Merck), EGFR-inhibitor gefitinib (LC Laboratories, Woburn, MA, USA), ERK1/2-inhibitor SCH772984 (Hycultec, Beutelsbach, Germany), MEK1/2-inhibitor U0126 (Selleckchem), cytostatics doxorubicin (Adrimedac, Wedel, Germany), or mitoxantrone (Hexal AG, Holzkirchen, Germany). In the case of combination effects, cells were seeded out in 80 µL medium. Afterwards, 8 concentrations (A-H) of substance A and 6 concentrations (1–12) of substance B were added in duplicates.

After incubating for 72 h, 20 µL of MTT solution (5 mg/mL) was added for 1 h at 37 °C and 5% CO_2_. After removing the supernatant, formazan was solubilized in 200 µL (MCF-7) or 100 µL (MDA-MB-231) of DMSO (Carl Roth, Karlsruhe Germany). Plates were analyzed using a plate reader (Thermomultiscan EX, Thermo Fisher Scientific Inc) at 570 nm, with background subtraction at 690 nm. Data were normalized to DPBS as 100% viability and absorption of zero as 0% viability and evaluated by a nonlinear regression (three or four parameters depending on which is the statistically favored one) to obtain pEC_50_ values of sigmoidal dose–response curves (GraphPad Prism 6, San Diego, CA, USA) [68]. Those values were used to calculate means, from which a mean EC_50_ value was calculated. In order to estimate whether the effect is synergistic, antagonistic or additive we used the Chou-Talalay method [69]. The resulting combination index (CI) was calculated by software CompuSyn^©^ (ComboSyn, Inc., Paramus, NJ, USA). A CI < 1 represents a synergistic effect, CI > 1 displays antagonism and CI = 1 show additive effects.

### 4.5. Proteome Profiler Array

Proteome Profiler™ Human Phospho-Kinase Array and Nonhematopoietic array Kits (both from R&D Systems GmbH, Wiesbaden-Nordenstadt, Germany) were performed to screen MCF-7 and MDA-MB-231 cells for changes in intracellular signaling pathways comparing untreated cells with the effects of COL1-binding and ITGB1-kd. Cells were grown to a confluence of 80–90% before lysis. Cell lysates were prepared and assay was performed according to the manufacturer’s protocol, a Pierce™ BCA Protein Assay Kit (LifeTechnologies, Thermo Fisher Scientific Inc) was used to quantify total protein. Membranes were photographed and quantified using ChemiDoc XRS+ imaging acquiring system (BioRad, Munich, Germany), and “Image Lab” software v.6.0 (BioRad). Uncut pictures of arrays are shown in Figure S5.

### 4.6. Western Blot

Cells extraction, lysate quantification, SDS-Page and Western blot were performed as described previously using stainfree gels [22]. Membranes were incubated with mouse anti-GAPDH (GeneTex, Irvine, CA, USA), mouse anti-pEGFR (Tyr1068, 15A2), mouse anti-BCRP (BXP-21), mouse anti-pHSP27 (Ser82, B-3), mouse anti-HSP27 (F-4), mouse anti-integrin β_4_ (A9), rabbit anti-ERK1/2 (Cell Signaling Technology, Frankfurt am Main, Germany), rabbit anti pERK1/2 (Thr202/Tyr204, Cell Signaling Technology) rabbit anti-DDR1 (Cell Signaling Technology), rabbit anti-pDDR1 (Tyr513, Cell Signaling Technology) as well as goat anti-rabbit and anti-mouse IgG kappa binding protein IgG HRP-conjugated diluted in 1% BSA solution. If not indicated otherwise, antibodies were purchased from Santa Cruz Biotechnology. Western blot was quantified via chemiluminescence using Clarity Western ECL substrate chemiluminescence kit (BioRad). Besides the loading control GAPDH, we used stainfree total protein normalization. Membranes were photographed and quantified using ChemiDoc XRS+ imaging acquiring system (BioRad) and Image Lab software v. 6.0 (BioRad). Representative uncut Western blots are shown in Figure S5.

### 4.7. Annexin V/Propidium Iodide

Apoptosis was analyzed using an Annexin V-FITC Apoptose Kit (eBioscience, Thermo Fisher Scientific) and a Guava^©^ flow cytometer (Merck). Cells were grown 24 h to a confluence of 60–70%. SCH772984 was added to a concentration of 0.316 or 1 µM and incubated for 48 h. Afterwards, cells were detached from the cell culture flasks by using 0.02% EDTA/Trypsin and incubated with either Annexin V-FITC and/or propidium iodide. Both dyes were excited at 448 nm and emission was measured in green (Annexin V-FITC) and red (propidium iodide) channel.

### 4.8. Functional Transporter Assay

In order to investigate the activity of ABC transporters, dye accumulation assays were performed. Cells were cultured 72 h in the presence or absence of COL1 up to 60–70% and/or 24 h in the presence or absence of ERK1/2-inhibitor SCH772984 (1 µM), detached using trypsin/EDTA (Pan Biotech) and suspended in Krebs-HEPES-Buffer (KHB). 40,000 cells were added per well of a 96-well plate. For BCRP transporter investigation, its substrate dye pheophorbide A (Frontier Scientific Inc., Logan, UT, USA) was used at a concentration of 0.5 µM. To obtain a total BCRP-inhibition, the inhibitor Ko143 (IC_50_ 0.276 µM [70], Tocris Bioscience, Bristol, UK) was used at a concentration of 3 µM. After 2 h incubation, fluorescence (λex = 405 nm, λem = 695 ± 50 nm) was measured by flow cytometry (Guava^®^, Merck). The values of KHB were normalized to each inhibited sample to get the functional transporter activity as a ratio to maximal inhibition. To analyze the structural effect of the inhibitors used, BCRP overexpressing MDCK II BCRP were used [70]. In these terms, inhibitors were added directly without any incubation.

### 4.9. Statistical Analysis

Comparisons were performed using the software Prism™ (GraphPad Software, San Diego, CA, USA). MTT results were analyzed by nonlinear regression (three or four parameters depending on which is the statistically favored one) to obtain sigmoidal dose–response curves and to determine the EC_50_ at the curves inflection point. Moreover, statistical analysis was performed using Student’s paired *t*-test (* *p* < 0.05; ** *p* < 0.01; *** *p* < 0.001). In combination approaches, *t*-test was used in order to compare concentrations of component B only to DPBS.

## 5. Conclusions

Our data confirm that the COL1 induced resistance of BCCs is mediated by either ITGB1 or DDR1 via activating the MAPK pathway, which offers promising targets for either treating the cells with signaling inhibitors in a combined manner, or to sensitize the cells for cytostatics, such as mitoxantrone or doxorubicin. The indicated cells differ in their cellular response since MDA-MB-231 as a model for TNBC strongly upregulate EGFR and MAPK signaling in these terms favoring a combined treatment including EGFR-inhibitors, such as gefitinib, as chemosensitizer. This study stresses the role of DDR1 as a therapeutic target as well as a potential biomarker for chemoresponse. Future in vivo assays are needed to confirm the efficacy of the indicated combinations.

## Supplementary Materials

The following are available online at https://www.mdpi.com/1422-0067/21/14/4956/s1, Figure S1. Annexin V/PI assay. Figure S2. Combination effect of 7rh and SCH772984 cytotoxicity in MCF-7 cells. Figure S3. The impact of ERK1/2, MEK1/2 and DDR1-inhibitors on the cytotoxic treatment of MDA-MB-231 and MCF-7 cells with doxorubicin or mitoxantrone in combinatory approaches. Figure S4. (a) Survival of MCF-7 cells in the presence of gefitinib in dependence of SCH772984. (b) Survival of MCF-7 and (c,d) MDA-MB-231 cells in presence of mitoxantrone or doxorubicin in dependence of EGFR inhibitor gefitinib Figure S5: Representative uncut Western blots and proteome profiler arrays, which are partly shown in Figure 1, Figure 2, Figure 3, Figure 4, Figure 5, Figure 6 and Figure 7.

## Abbreviations

ABCG2	ATP-binding cassette super-family G member 2/breast cancer resistance protein (BCRP)
AKT	protein kinase B
BC	breast cancer
BCC	breast cancer cell
CAM-DR	cell-adhesion-mediated drug resistance
DDR1	discoidin domain receptor 1
DMEM	Dulbecco’s Modified Eagle’s Medium
DMSO	dimethyl sulfoxide
DPBS	Dulbecco’s Phosphate-Buffered Saline
ECM	extracellular matrix
EGFR	epidermal growth factor receptor
ERK1/2	extracellular signal-regulated kinases 1 and 2
Grb2	Growth factor receptor-bound protein 2
HER2	human epidermal growth factor receptor 2
HSP27	heat shock protein 27
ITGB1	integrin β_1_
ITGB4	integrin β_4_
kd	knockdown
MAPK	mitogen-activated protein kinase
MEK1/2	MAPK/ERK kinase 1 and 2
p	phosphorylated
RTK	receptor tyrosine kinase
sc	scrambled
SOS	Son OF Sevenless
TNBC	triple-negative breast cancer
wt	wildtype
