# Supplementary material for: Targeting Discoidin Domain Receptor 1 (DDR1) Signaling and Its Crosstalk with β1-Integrin Emerges as a Key Factor for Breast Cancer Chemosensitization upon Collagen Type 1 Binding
Source: Int J Mol Sci. 2020 Jul 13;21(14):4956. doi: 10.3390/ijms21144956 (PMC7404217; doi:10.3390/ijms21144956)
Supplement: Supplementary file 1 [file ijms-21-04956-s001.pdf]

International Journal of
Molecular Sciences

MDPI

Supplementary Materials

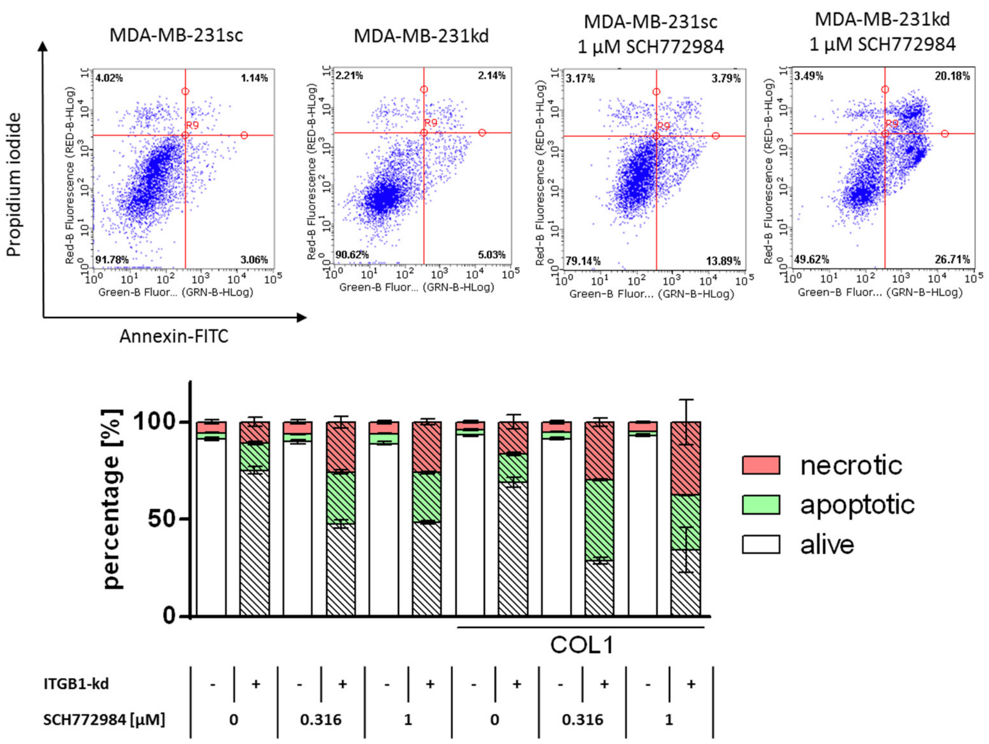

Figure S1. Annexin V-FITC/Propidium iodide assay of SCH772984 (0.316 µM and 1 µM, for 48 h) incubated MDA-MB-231 scrambled (sc) and integrin β1-knockdown (ITGB1-kd) cells in the presence and absence of collagen type 1 (COL1, *n* = at least 3 biological samples).

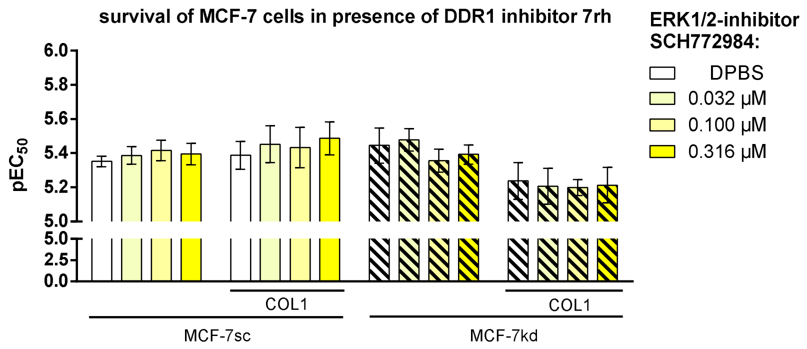

Figure S2. Combination effect of 7rh and SCH772984 cytotoxicity in MCF-7 scrambled (sc) and integrin β1-knockdown (kd) cells in the presence and absence of collagen type 1 (COL1), displayed by the pEC_{50} values of 7rh cytotoxicity in dependence of SCH772984 (*n* = at least 3 biological samples). MCF-7 cells showed no sensitization upon this combination treatment. Statistical analysis was performed via unpaired t-test.

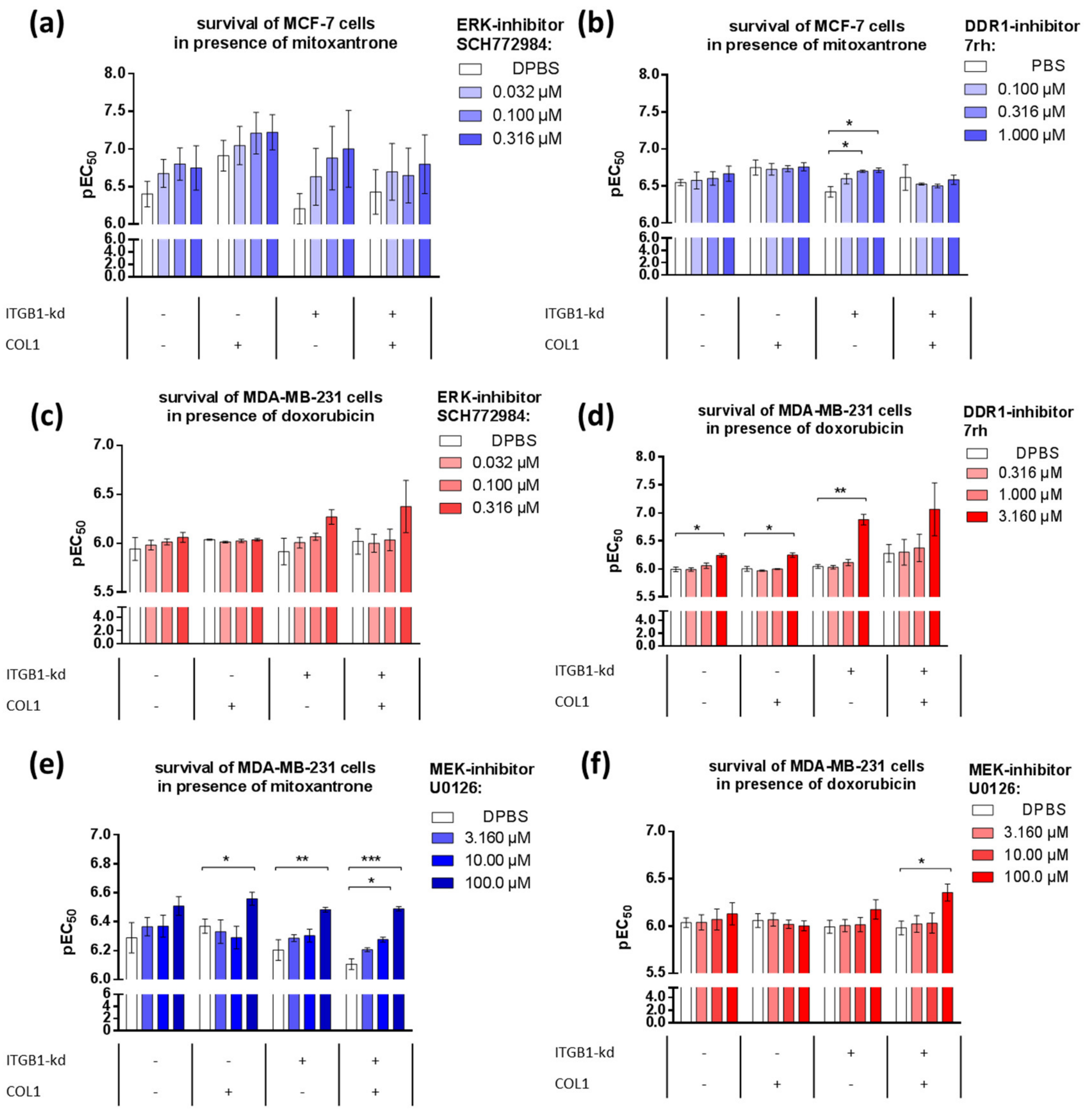

Figure S3. The impact of ERK1/2, MEK1/2 and DDR1-inhibitors on the cytotoxic treatment of MDA-MB-231 and MCF-7 scrambled and integrin β1-knockdown (ITGB1-kd) cells with doxorubicin or mitoxantrone in combinatory approaches in the presence and absence of collagen type 1 (COL1). **(a,b)** The ERK1/2-inhibitor SCH772984 **(a)** and DDR1-inhibitor 7rh (**b**) were combined with mitoxantrone in MCF-7 cells. **(c,d)** The ERK1/2-inhibitor SCH772984 **(c)** and DDR1-inhibitor 7rh **(d)** were combined with doxorubicin in MDA-MB-231 cells. **(e,f)** The MEK1/2-inhibitor U0126 was combined with **(e)** mitoxantrone and **(f)** doxorubicin in MDA-MB-231 cells. The cytostatics were applied in increasing concentrations to gain a pEC_{50} value. The inhibitors were additionally added at a constant concentration. Shown are means of pEC_{50} values and statistical analysis was performed using unpaired t-test (*$p < 0.05$; **$p < 0.01$, $n = 3$ biological samples).

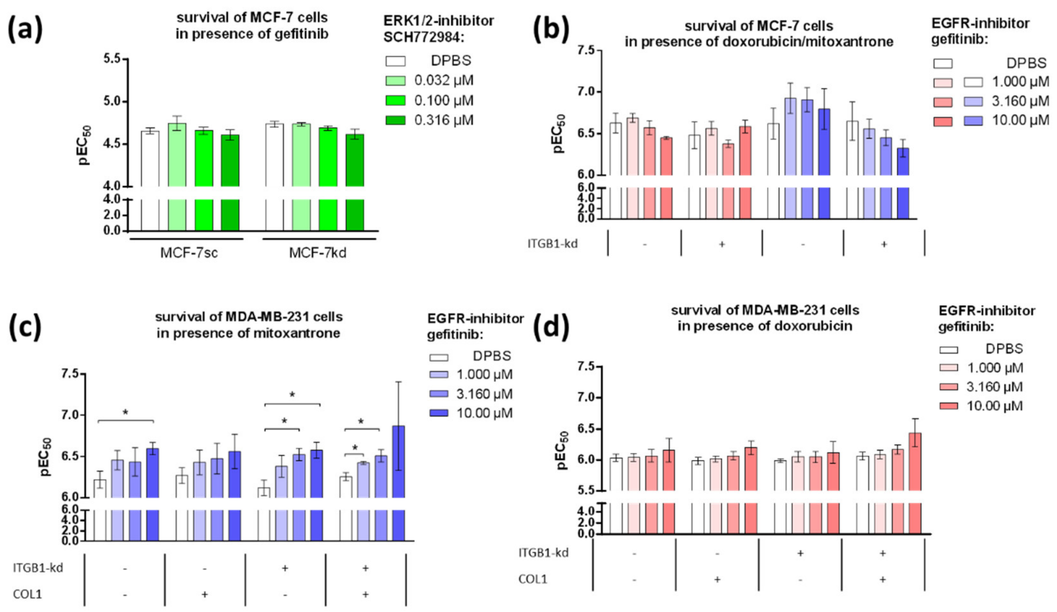

Figure S4. **(a)** Survival of MCF-7 scrambled (sc) and integrin β_1-knockdown (ITGB1-kd) cells in the presence of gefitinib in dependence of SCH772984. Inhibition of EGFR by gefitinib had no further synergistic effect with ERK1/2-inhibition in MCF-7 cells. **(b)** Survival of MCF-7 and **(c,d)** MDA-MB-231 sc and kd cells in the presence and absence of collagen type 1 (COL1). The cells were incubated with mitoxantrone or doxorubicin in dependence of EGFR inhibitor gefitinib. Statistical analysis was performed via unpaired t-test (n = at least 3 biological samples, $*p < 0.05$).

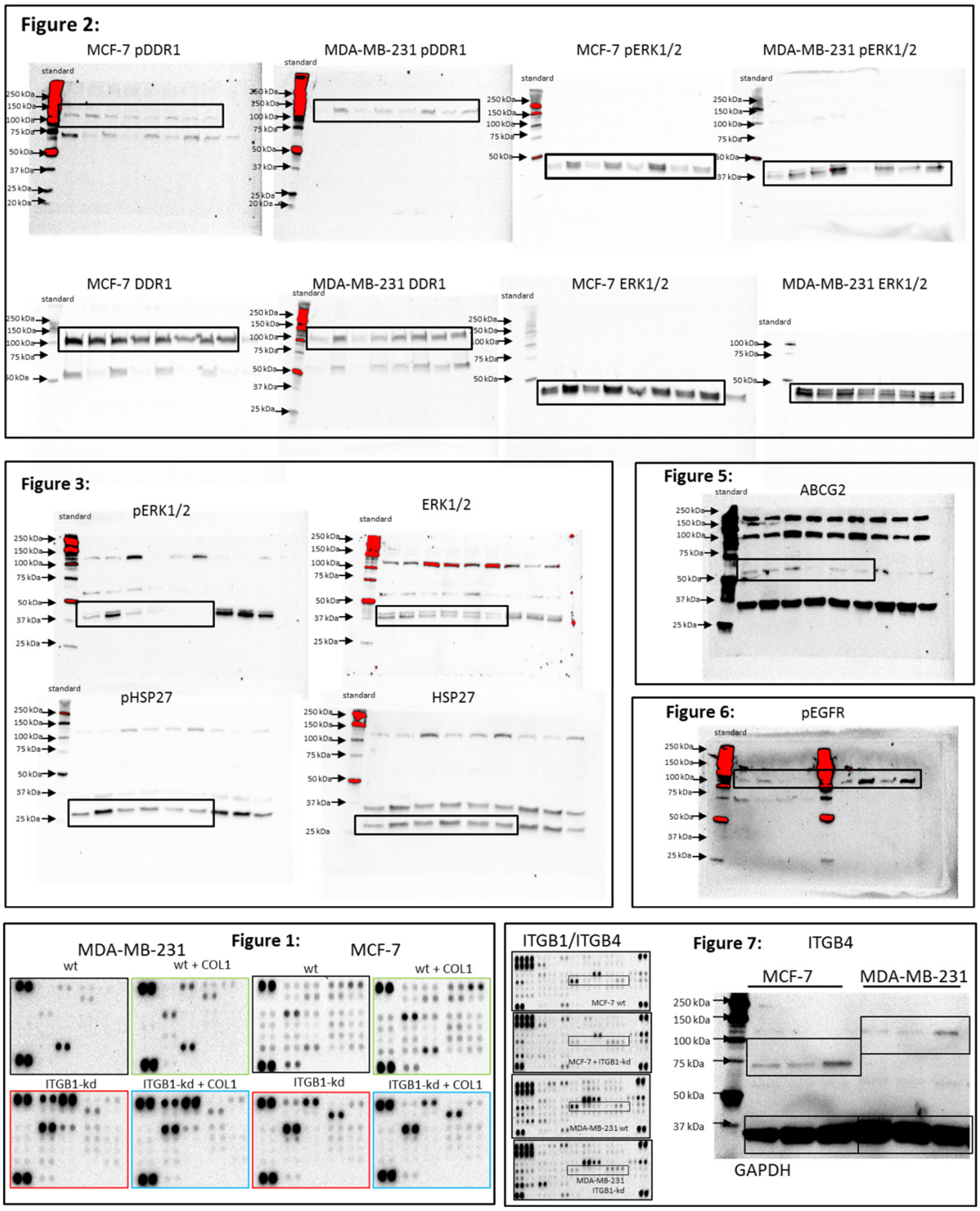

Figure S5: Representative uncut Western blots and proteome profiler arrays, which are partly shown in Figures 1–7.

© 2020 by the authors. Licensee MDPI, Basel, Switzerland. This article is an open access article distributed under the terms and conditions of the Creative Commons Attribution (CC BY) license (http://creativecommons.org/licenses/by/4.0/).
